# Identifying unique barriers to implementing rural emergency department-based peer services for opioid use disorder through qualitative comparison with urban sites

**DOI:** 10.1186/s13722-022-00324-3

**Published:** 2022-07-28

**Authors:** Dennis P. Watson, Monte D. Staton, Nicole Gastala

**Affiliations:** 1 Chestnut Health Systems, 221 W. Walton St, Chicago, IL 60610 USA; 2 Center for Dissemination and Implementation Science, Department of Medicine, University of Illinois College of Medicine at Chicago, Chicago, IL USA; 3 Mile Square Health Centers, Department of Family Medicine, University of Illinois College of Medicine at Chicago, Chicago, IL USA

**Keywords:** Peer services, Overdose, Substance use disorder, Behavioral health services, Program implementation, Implementation context

## Abstract

**Background:**

In an effort to address the current opioid epidemic, a number of hospitals across the United States have implemented emergency department-based interventions for engaging patients presenting with opioid use disorder. The current study seeks to address gaps in knowledge regarding implementation of a sub-type of such interventions, emergency department-based peer support services, in rural areas by comparing implementation of rural and urban programs that participated in Indiana’s Recovery Coach and Peer Support Initiative (RCPSI).

**Methods:**

We conducted a secondary analysis of qualitative semi-structured implementation interviews collected as part of an evaluation of 10 programs (4 rural and 6 urban) participating in the RCPSI. We conducted interviews with representatives from each program at 3 time points over the course of the first year of implementation. Our deductive coding process was guided by the Consolidated Framework for Implementation Research (CFIR) and an external context taxonomy.

**Results:**

We identified key differences for rural programs corresponding to each of the 5 primary constructs in the coding scheme. (1) Intervention characteristics: rural sites questioned intervention fit with their context, required more adaptations, and encountered unexpected costs. (2) External context: rural sites were not appropriately staffed to meet patient needs, encountered logistical and legal barriers regarding patient privacy, and had limited patient transportation options. (3) Inner setting: rural sites lacked strong mechanisms for internal communication and difficulties integrating with pre-existing culture and climate. (4) Characteristics of individuals: some rural providers resisted working with peers due to pre-existing attitudes and beliefs. (5) Implementation process: rural sites spent more time identifying external partners and abandoned more components of their initial implementation plans.

**Conclusions:**

Findings demonstrate how rural programs faced greater challenges implementing emergency department-based peer services over time. These challenges required flexible adaptations to originally intended plans. Rural programs likely require flexibility to adapt interventions that were developed in urban settings to ensure success considering local contextual constraints that were identified by our analysis.

## Background

With 446,032 overdose deaths attributed to opioids from 1999 to 2018 [1], the opioid epidemic is one of the most serious public health issues facing the United States. While a national problem, some researchers have argued the burden of the epidemic is much greater in rural communities [2], which is partially due to rural areas having fewer and less easily accessible opioid use disorder (OUD) treatment options [3]. Despite a recognized need to improve understanding of rural opioid use and treatment [4], a recent scoping review identified relatively few studies on this topic [2]. The study described in this article identifies implementation differences across 4 rural and 6 urban hospital systems participating in Indiana’s Recovery Coach and Peer Support Initiative (RCPSI), which supported delivery of novel emergency department (ED)-based peer support services. Identifying differences in implementation for ED-based peer supports is timely considering the recent national proliferation of similar interventions [5, 6].

The RCPSI was a federally funded initiative that supported the integration of peer services to engage ED patients presenting with OUD and link them to treatment and services [7]. The initial idea for the RCPSI was partially inspired by peer support programs developed for patients presenting with OUD in urban EDs [8, 9]. While a small but developing body of research supports the potential effectiveness of ED-based peer supports for improving outcomes for people with OUD [8,10], no recognized standard for the implementation of these programs currently exists. However, prior research has identified three core functions of such programs [6]: (1) peers are somehow integrated into the ED environment; (2) patients presenting with OUD are identified and connected with peer supports; and (3) peers connect patients with medication for opioid use disorder (MOUD) or other treatment services and supports. Implementation of these three core functions vary depending on the ED context [6]. However, the ways in which these contextual differences impact implementation have not been explored in detail. Furthermore, we are aware of only two studies of ED-based OUD interventions that have focused on the rural ED context [11,12] and both were focused on buprenorphine induction, rather than peer supports.  More rural studies of ED-based OUD interventions, including peer-facilitated ones, would be useful considering prior work has demonstrated that barriers unique to rural settings can limit the effectiveness of a variety of OUD interventions [13–17]. For instance, one of the most pervasive rural barriers likely to impact peers’ linkage function is a lack of MOUD treatment options [18–22]. Proactive identification of such barriers and their impacts on implementation success can lead to adaptations that can make an intervention more viable than if it was directly translated from an urban setting [23].

The current qualitative study addresses existing gaps in knowledge related to implementation of ED-based peer supports for OUD in rural areas. The primary questions guiding this study were: (1) what determinants (i.e., barriers and facilitators) most impacted ED-based peer service implementation?; and (2) how did these determinants differ between rural and urban sites over the course of implementation?

## Methods

We conducted a secondary analysis of data originally collected to evaluate Indiana’s RCPSI. Indiana University’s institutional review board determined the project did not require review because the data were originally collected for quality assurance purposes and were de-identified prior to secondary analysis.

### Description of the RCPSI and its funded sites 

Indiana’s Division of Mental Health and Addiction supported the RCPSI from September 2017 to May 2020 with funds from the U.S. Substance Abuse and Mental Health Services Administration. The RCPSI’s goal was to implement the peer position within EDs, rather than implement a defined program model. This is because no robustly defined evidence-based model for ED-based peer linkage programming existed at the time programs were funded and because the state authority recognized the need for agencies to have latitude during implementation due to their differing contexts. As such, the only specific requirements were that the peer (a) completed state-level peer recovery coach certification, (b) engaged OUD patients who presented to the ED, and (c) attempted to link those patients with MOUD or other appropriate treatments and supports.

Ten hospital systems participated in the RCPSI. These systems varied in size and number of participating EDs within them. The state provided funding directly to one primary vendor for each program, which could either be the hospital system itself or an external behavioral health provider who offered peer services (we refer to vendors rather than hospitals when discussing the involved systems moving forward, as this was the term used by the state agency that supported the RCPSI). Four of the vendors exclusively focused their RCPSI programming in hospitals with a rural critical access designation. While some of the larger systems served both rural and urban EDs, the implementation data focused considerably, if not exclusively, on their hospital sites located within cities. We therefore consider those vendors to be urban-serving for the purpose of this study. Regarding the 3 functions of ED-based peer supports discussed previously [6], (1) peer integration varied according to their physical location in relation to the ED, with only three programs having some sort of space within the ED for peers to work. (2) Identification of patients with OUD and activation of peer supports relied largely on ED staff; however, peers for four of the vendors could scan for appropriate patients using the electronic health record. (3) The goal of all programs was to connect patients to MOUD or other treatment, but supportive services were offered to those who were not yet ready. Table 1 displays characteristics of the vendors’ hospital systems and the acronyms used to identify them. It is important to note that, while they participated in evaluation activities, RV4 did not follow-through with implementation of the program; though, they were able to speak to the barriers influencing this result.

**Table 1:** RCPSI vendor identifiers and characteristics

Vendor identifier	Vendor relationship to the hospital system served	Single or multiple ED(s) served*	Peer physical location	Identification and activation of peers
Rural Vendor 1 (RV1)	Same	Single	On-site office outside ED	ED staff initiate
Rural Vendor 2 (RV2)	Same	Single	On-site office outside ED	ED staff initiate
Rural Vendor 3 (RV3)	Same	Single	Off-site with no on-site space	ED staff initiate
Rural Vendor 4 (RV4)	Same	Single	n/a	n/a
Urban Vendor 1 (UV1)	External provider	Multiple	Primarily off-site but have on-site ED space to work	ED staff initiate
Urban Vendor 2 (UV2)	Same	Multiple	On-site office in ED	ED staff initiate & peer monitoring of electronic health record
Urban Vendor 3 (UV3)	Same	Multiple	Off-site with no on-site space	ED staff initiate & peer monitoring of electronic health record
Urban Vendor 4 (UV4)	External provider	Single	Off-site but have on-site ED space to work	ED staff initiate & peer monitoring of electronic health record
Urban Vendor 5 (UV5)	Same	Multiple	On-site office outside ED or off-site depending on hospital	ED staff initiate & peer monitoring of electronic health record
Urban Vendor 6 (UV6)	Same	Multiple	Off-site with no on-site space	ED staff initiate

*Detail related to the exact number of EDs served is not shown to limit the degree to which vendors could be identified

### Data collection

Data collection occurred between April 16, 2018 and March 8, 2019. Each vendor participated in three semi-structured interviews that occurred at the beginning (T1), mid-point (T2; 4–6 months depending on vendor) and near the end of the first year (T3; 9–11 months depending on vendor) of implementation respectively. Vendors provided the name and contact information of a primary evaluation liaison to help arrange interviews. The number of interview participants ranged from 1 to 3 depending on the vendor and time point; however, interviews always included the primary person leading implementation at the site. Additional interview participants were typically supervisors of the vendor’s peers. Peers were only involved in meetings at UV3 & UV4. In the case of UV3, the peer took over as the primary implementation lead after the original person in this role left the organization. While UV3’s peers joined T2 & T3 meetings, the main contributor to the conversation was the implementation lead.

Evaluation data collection was led by the first author who is a trained qualitative researcher and implementation scientist with experience working on several opioid treatment studies. He was assisted by two staff researchers with advanced training in qualitative methods and program evaluation. Questions were adapted from those developed from the Consolidated Framework for Implementation Research (CFIR), which organizes 37 constructs reflective of implementation determinants across five domains [24]: (1) the intervention’s defining characteristics, (2) the inner setting (i.e., environment in which the intervention is being implemented), (3) the outer setting (i.e., the environment existing outside of the implementing organization), (4) characteristics of individuals involved in the implementation, and (5) the process that facilitated the implementation. Therefore, each vendor was asked the same series of questions related to the 5 CFIR domains. The first interviews occurred in person at vendors’ sites to establish familiarity between evaluations and local leaders, and subsequent interviews occurred over conference call. All interviews were recorded and lasted between 16 and 63 min. As funding for the project supported interview participants’ time involved in evaluation activities, they were not separately compensated for interview participation.

### Analysis

We followed a case study approach [25, 26] to analysis with the vendors being the main unit of analysis (i.e., cases). The number of cases exceeds the minimum of four recommended for such analyses [25]. While the data demonstrate valuable information related to implementation in both rural and urban contexts, the analysis concentrated on understanding how rural sites differed. This decision was based on the need to focus the analysis given (a) rural vendors’ discussions suggested more difficulties with implementation and (b) the lack of attention to rural sites in the existing literature.

Analysis was organized with MAXQDA software [27]. The first part of the process was deductive and used an a priori coding structure developed from two sources (see Hanna et al. [28] for a prior example of such an approach). The first source was the CIFR [29]. The second was a taxonomy of external context constructs [30], which comprises 8 constructs and was substituted for the CFIR’s outer setting domain. The reason for this substitution is because the CFIR was developed within a relatively closed clinical setting, while the external context taxonomy pulled from a variety of clinical and public health interventions. Therefore, the taxonomy provides more nuances related to the context outside of a hospital’s walls, which was important given the RCPSI vendors relied heavily on state and local resources and because our goal to compare rural and urban programs requires a detailed look at the community context they are set within.

A second analyst, a medical doctor with expertise in rural MOUD implementation, reviewed the codes, indicating areas of disagreement, while also applying a second-level code indicating whether the construct represented an implementation barrier or facilitator. The lead analyst then reviewed the codes again and discussed areas of disagreement with the second analyst until 100% consensus was met. In the second part of the analysis, the lead analyst attributed each hospital system with a rural or urban destination. He then used MAXQDA’s interactive quote matrix function to explore codes (1) within and across cases and then (2) within and across rural and urban hospital groups.^1^ Because of the large number of codes and to ensure determinants identified were associated with a rural context, rather than just a single hospital, the analysts focused on only those constructs reflecting data from at least two rural hospitals. The final step in the analysis was inductive, with two analysts identifying and establishing themes within the CFIR constructs through a consensus process and identifying how they differed between the urban and rural vendor groups over the course of the implementation (e.g., T1, T2, & T3 data). We determined saturation to be at the point when no additional insights were gained through further iterations between data and developing themes because adding new cases or interviews was not possible [25].

## Results

Findings demonstrating differences between rural and urban EDs are presented below by 4 of the CFIR domains, with the external context taxonomy substituted for the CFIR’s outer setting domain. Specific constructs are italicized within the paragraphs. Table 2 displays the themes categorized by domain and construct, which are also defined.

**Table 2:** Definitions and source for implementation domain and construct names identified in the analysis

Domain	Construct	Primary issues identified
Intervention Characteristics^a^		
	Evidence, strength, and quality	Awareness of successful pre-existing ED-peer influenced adoption decisions for all vendors, but rural vendors were skeptical about applicability to their settings.
	Intervention source	
	Relative advantage	Rural vendors saw advantages of peers outside of the ED, where urban vendor solely focused on peer advantage in the ED.
	Adaptability	Rural sites made more adaptations over time to address peers’ low work volume.
	Cost	Rural vendors had greater concerns regarding costs.
External context^b^		
	Target population	Rural vendors did not have resources to fully staff peak overdose admission times or the ability to engage with transferred patients. Patients in rural areas also tended to use drugs other than opioids.
	Relational climate	Rural vendor slacked protocols to follow-up with transferred patients.
	Policy and legal climate	Privacy laws limited rural vendors' ability to share information with other hospitals where patients were transferred.
	Local infrastructure	Rural areas lacked treatment providers for patient referral and options for transportation to referrals were limited.
Inner setting^a^		
	Networks and communication	Rural vendors lacked strong mechanisms for communication between ED staff and peers. Rural providers were often reluctant to have peers see patients.
	Culture	Rural peers frequently encountered lack of respect for their lived experience and negative attitudes toward addiction on the part of ED staff.
	Implementation climate	Rural vendor experienced difficulties justifying integration of peer services into ED systems and workflows due to low volume of patients eligible for peer services.
Characteristics of individuals^a^		
	Knowledge and beliefs	Rural providers resisted working with peers and patients they served due to pre-existing beliefs.
Implementation process^a^		
	Engaging	Rural vendors had to spend more time identifying external providers to refer patient to. They also spent more time engaging local law enforcement in order to create more work for peers.
	Executing	Rural vendors abandoned more components of their initial implementation plans because of staff resistance and low patient volume.

^a^Source: Consolidated Framework for Implementation Research [29]

^b^Source: External context taxonomy [30]

### Intervention characteristics

The perceived *evidence* supporting peer services and the *source* of pre-existing ED-based peer programs was an important facilitator of adoption discussed at T1 interviews. Vendors described awareness of research on peer supports as influencing their decisions to apply for RCPSI funding. For instance, a representative from RV1 spoke about research they looked to when considering applying:

I did a little research on it [peer services], and it made a lot of sense. It works for AA [Alcoholics Anonymous] and NA [Narcotics Anonymous] to get people who’ve been through it [12-step programming] to be your coach and support [i.e., sponsor]. But, then this [peer services in the ED] takes it to another level because they’re [the peers are] more involved with connecting to the [treatment] resources around you [in the community]. (T1)

While the above statement applies logic from other types of peer supports to the decision to implement ED-based peers, other vendors directly discussed knowledge of early successes of ED-based peer support programs they had learned about as influencing their decisions. While urban vendors discussed similar reasons for applying for the RCPSI funding at T1, a key difference was that some urban vendors (e.g., UV3, UV4, and UV6) physically visited sites that had implemented ED-based peers to learn directly from them. While all vendors were aware of early successes of other ED-based peer programs, rural vendors were the only ones that displayed skepticism about the ability to translate these services directly to their settings. As noted by one rural vendor, “[a] lot of this research [on ED-based peers] is only hypothetically applicable to rural [areas]…” (RV2; T1).

Across vendors, the addition of peer services was viewed as having a *relative advantage* compared to usual care provided to OUD patients presenting in their EDs. This is because most hospitals did very little for these patients after ED discharge: “Honestly…[prior to applying for RCPSI funding] we do not do anything post ED [discharge]” (RV4; T1). As such, vendors viewed peer supports as a valuable resource for patients that would provide needed relief to ED staff: “at this point [T1], the physicians and nurses do what they can. They just see it [treating OUD patients] as too much for them, so they are eager to get additional resources [from the peers]” (UV2). However, as the implementation progressed, urban vendors continued to discuss relative advantage as it related to the ED environment, while rural vendor discussions shifted to described how the advantage of peer services extended beyond the ED’s boundaries. For instance, data from RV2 demonstrated they were using the peer to fill a need for outpatient services: “We had a great need for peer recovery coaching in the outpatient side. So, [the peer has] actually been helping with our outpatient addictions program too, and then just coming over here [to the ED] when called” (T3).

Interviews also demonstrate the high degree of *adaptability* of peer services. While all vendors discussed ways in which the peer position was different from those on which the RCPSI was partially based and how the peer role evolved over time, rural vendors’ discussions demonstrated they made more considerable modifications to the scope of peer services in an effort to address the fact that peers had very little work due to low numbers of OUD patients being admitted to the ED. The previous quote from RV2 demonstrates how their peer’s scope was extended to include outpatient services, and an earlier interview also showed how the peer was working with other departments: “[our peer] also does [work in] the OB [obstetrics department], and she also is involved with our court program [for justice-involved patients]” (T2). In another example, RV3 contracted with a local off-site mental health provider that designated a peer to respond to the ED when needed since it did not have enough patients presenting with OUD to justify employing a full-time ED-based peer.

Rural vendors found the *costs* related to peer services to be a greater barrier at all interview points. Early in the implementation, RV1 and RV4 both expressed concerns for how they would support peers because they did not have enough target patient volume to cover the salaries. However, this was demonstrated to be resolved at T2 when the funding model was changed from service reimbursement to one that directly covered peer salaries. At T1, RV2 had concerns about the costs getting “completely out of control” because their hospital had only one part-time, on-call psychiatrist available who warned they were overwhelmed with their peer’s referrals. It was noted in later interviews that the administration hired a full-time psychiatrist to address this issue, which was an unexpected cost at the time they applied for RCPSI funding. The cost of technology also later derailed RV2’s original plans to implement telehealth-based peer services as an addition to their program, as they had no monies left to do this after using RCPSI funds to update their electronic health record to document peer service contacts. In contrast, urban vendors directly stated they had no or very little concerns regarding implementation costs, and they did not discuss any unexpected costs to have occurred over the course of implementation.

### External context

*Target population* needs greatly impacted peer service feasibility at some rural sites. Specifically, OUD patients’ needs that were not anticipated at baseline resulted in missed engagement opportunities and low peer caseloads. One aspect of this was that rural vendors did not have resources to cover evening hours when many overdoses presented to the ED. As an example, RV2 and RV3 peers missed patients due to restricted hours, with RV3 stating in their final interview that no overdose patients had been admitted to the ED when a peer was available. Another unexpected issue was that rural hospitals often transported overdose patients to urban hospitals that could provide services for which rural vendors were unequipped to handle. Because these patients were often unconscious prior to transfer, peers were unable to engage them or obtain a release required to speak about their case to staff at the hospital to which they were transported: “For what we see in our emergency room, [overdose patients are] either very ill, intubated, [or] transported to a higher level of care [at another location]” (RV3; T3). Finally, rural vendors also expressed in later interviews that the majority of patients who could benefit from peer services had other substance use disorders, which were outside of the RCPSI’s scope: “We have a strong uptick in stimulants here in this part of the world…I suspect that’s a big reason why we’re not seeing a lot of [opioid overdoses]” (RV2; T3). While urban vendors also noted the need to serve patients with other substance use disorders, enough OUD patients presented to the ED to make up a considerable portion of peer caseloads.

Connected to the above issue of patient transportation to urban hospitals are factors associated with the external *relational climate*, which includes the quality of relationships with other hospitals and providers. Despite rural hospitals having relationships and procedures necessary to transport patients to urban hospitals, they had not developed protocols that would allow peers to follow-up regarding a patient’s status. This lack of information sharing was a major barrier to implementation for rural hospitals because peers needed to communicate with patients to link them with treatment and services. As an example, RV3 expressed how their peers had no way of knowing if the patient was transferred to another hospital or if they are “gonna come back in our community” (T2) where they should be connected to local resources by the peer. Information sharing with other health providers is also a matter of the *policy and legal climate*, as this sharing is governed by external regulations, including rules, policies, and laws, that impact implementation. This was not an issue in urban hospitals, which had the resources necessary to address most OUD patients’ care needs internally.

Rural vendors also described their *local infrastructure* as lacking needed OUD services and resources. While urban vendors also described such issues, it was far more detrimental for rural ones. For instance, where urban vendors discussed issues finding transportation assistance to help patients attend referral appointments, rural vendors did not even have sufficient providers in their local areas to which they could refer patients. Indeed  most rural vendors  had to rely on a single MOUD prescriber, and this was a concern for them over the entire course of implementation. For instance, at T1 RV3 expressed concern about their sole MOUD prescriber who they felt was already overwhelmed with referrals. In the most extreme case, RV4 was not able to find any MOUD provider to work with them: “that was my biggest hurdle because I did not have access to a nurse practitioner or anyone [to prescribe MOUD]” (T3). This was a major factor leading to RV4’s not following through with RCPSI implementation.

### Inner setting

*Networks and communication* among inner setting actors were key to implementation for all sites, with the fundamentally important line of communication demonstrated to be the one existing among peers and the ED’s medical staff. It is through this communication that peers are notified if and when they can approach an ED patient. All vendors had some growing pains establishing initial lines of communication; however urban vendors describe minimal issues or had largely solved any recognized problems by their final interview. For instance, at baseline, UV1 discussed their plan for integrating peers in the ED but described difficulties getting ED staff to understand that communicating with the off-site peers was not a violation of patient confidentiality at T2. However, this issue was largely solved by T3. Regarding communication issues at rural sites, RV1’s ED staff were supposed to call the peer when an appropriate patient presented. Though, they never identified a mechanism to ensure the peer was alerted. This resulted in the peer needing to “make more of an effort” (T2) to identify patients without ED staff assistance. Likewise, RV2 interviews demonstrated difficulty developing effective lines of communication between the ED staff and peer: “they [ED staff] would kind of just stare at her [the peer] and not talk to her” (T2). It was further explained that RV2’s ED staff would contact the social workers when OUD patients presented instead of alerting the peer. For all three rural vendors who carried through with implementation, they discussed these communication issues as persisting through their final interviews.

Differences in ED *culture* were highly noticeable when comparing rural and urban vendors. This was most apparent when it came to attitudes and actions of ED staff toward behavioral health care workers, including the peer. RV2 explained “[The ED staff] like things a certain way, and it’s hard to fit behavioral health into that box sometimes…This has been a historical thing for the hospital” (T2). RV3 also described a “bias of [i.e., against] addiction” (T3) among ED staff in which they hesitate to contact the peer because they do not believe there is anything that can be done to benefit overdose patients. By contrast, site leaders of urban vendors largely reported more welcoming ED staff attitudes, with the largest issue being the introduction of the peer role and its fit within the current ED hierarchy: “in the medical realm, the more initials you have behind your name, the higher status you have…Many of them [peers] don’t have any letters behind their names…but I feel like they’ve gained and earned respect within our health system” (UV5, T3).

The *implementation climate* was another important factor. Shared receptivity for RCPSI programming was lacking at RV2, where implementation leaders struggled to put necessary processes in place with the finance department and ED because there was a “resistance to behavioral health” (T2) that resulted in difficulties integrating peer services into pre-existing systems and workflows, and this issue was not fully solved by T3. RV3 attribute ED staff implementation resistance to the fact that they could not “hardwire” (T3) peer services into the pre-existing workflow since OUD patient volume was too low to justify a need for change among ED staff. In contrast, urban vendors and hospitals displayed more capacity for change (particularly regarding technological change), receptivity to peer services, and positive expectations about them among administration, ED staff, and behavioral health staff of involved organizations. As an example, UV2’s ED physicians were stated to be “very eager to hear [about the program]. They were interested in Narcan [the opioid overdose reversing drug] being used, they were very happy to hear about the [MOUD] clinic opening up and then, to hear about actually having a coach [i.e., peer] on site” (T1) from time they first learned about the peer services.

### Characteristics of individuals

ED staff members’ *knowledge and beliefs* about peer services resulted in barriers to implementation initially for two urban (UV4 and UV6) and two rural (RV2 & RV3) vendors. In the case of the two urban sites, individual-level resistance receded over time. One interview with UV4 provides an example of something a physician said that highlights this: “[a physician said] I was really against this [peer supports] at first, but I kind of see this working, so I think I’m gonna try this with some of my patients” (T2). In contrast, individual-level physician resistance at the two rural sites persisted. At T2, an RV3 physician was described as being “reluctant to open up just to anybody and say that [he can prescribe] Suboxone [a band name formulation of the MOUD buprenorphine]” because he did not think he could meet the potential demand that would be created by the peer services. Additionally, RV2 “never could really get the [ED] staff on board with why you would call a peer recovery coach [i.e., peer] over an LCSW [Licensed Clinical Social Worker] when the LCSW has more training” (T3), suggesting they did not believe a peer could provide services of a similar quality.

### Implementation process

Both rural and urban vendors recognized the importance of *engaging* health providers in various roles, including ED staff and local MOUD providers. One already discussed difference for rural sites was that they lacked MOUD providers in their communities to whom they could refer patients. Due to this lack of physicians, RV3 and RV4 sought out providers who they could support to obtain training necessary to prescribe MOUD, something no urban vendors needed to do. Although both rural and urban vendors described a process of engaging with and winning over the ED staff, mainly nurses, rural vendors experienced more difficulty with this over the course of implementation: “ [the peer service program] hasn’t been accepted [by ED frontline staff] quite as well as I thought it would be in the beginning” (RV2; T2). Engaging external organizations was also important for peer implementation. Both rural and urban vendors disseminated information about their peer services to local physicians, law enforcement agencies, and community organizations; however, rural vendors were much more focused on law enforcement. For instance, RV2 and RV3 developed relationships with local drug courts and probation departments, with RV2 using this as a means of providing more work for their peer given the low patient volume in the ED previously discussed: “she [the peer] also does some case management for our people in our court program” (T2).

In *executing* the implementation, both rural and urban vendors described peer hiring challenges and difficulties incorporating them into the ED workflow, but overall, rural vendors were less successful following through, with RV4 discontinuing and RV2 still trying to gain ED staff cooperation by the end of the first year of programming. As previously stated, rural vendors experienced greater difficulty getting ED staff to alert peers when patients presented who were eligible for their services, as with RV3: “[for] My [ED staff], still, [the peer is] not top-of-mind there yet, and she’s gone down there and spoken to them, and shadowed them for a shift, a couple different things, but yeah, if anybody has any ideas on how to get more buy in from your [ED staff], I’d be interested” (T3). RV2 described great difficulty setting the electronic health record system up to track peer services, which was not a problem for any of the urban vendors who discussed requiring similar technological adaptations. While urban vendors also described some challenges executing the implementation, these discussions focused more on successes and their underlying facilitators, as they indicated having more support from ED staff, more helpful technology departments with better resources, and an easier time integrating peer services into the pre-existing workflow. A typical example of the smoother execution of the implementation plan among urban vendors is demonstrated by a selection from in UV4’s T3 interview where participants listed barriers they had encountered and overcome:

The first barrier was training up a group of recovery coaches [i.e., peers] that we could look at to hire. We overcame that barrier…a few times we had some issue with ‘Oh my gosh how’s the person [patient] gonna get their medication?’…but we were able to utilize [our foundation] and different things to help….

Such discussions of successful resolutions to major implementation barriers were not a feature of later rural vendor interviews.

## Discussion

Our secondary analysis of implementation interviews from the evaluation of the RCPSI identified a number of differences between rural and urban locations. In some instances, issues raised were completely unique to rural sites, such as the concern that peers might not be completely adaptable to the local context. This was likely rooted in the fact that the RCPSI, like most substance use interventions, was developed without explicit consideration of the rural healthcare context [31]. In other instances, similar issues impacted sites but differed in the degree of their perceived effect over time. For instance, all vendors viewed peers as filling a gap in both setting types, but rural vendors viewed them as filling this gap beyond the confines of the ED, largely due to their need to make adaptations in response to challenges encountered. The rest of this discussion focuses on the most salient and actionable themes identified.

One of the most unique and pressing issues for rural sites was the lack of patient referrals to peers, which is one of the core functions of such programs [6]. One unavoidable reason for this was the low volume of service-eligible ED patients. Compounding this issue, was the fact that peers did not work evenings when most eligible ED patients were likely to present. The data do not provide an exact reason for these restricted hours, but rural vendors did have smaller budgets that prevented hiring peers to provide more extensive coverage and scheduling peer shifts during normal business hours was likely to attract more potential hires in rural areas where the applicant pool was likely smaller. One potential solution to low patient volume and incompatible staffing coverage would be to partner with an external peer telehealth hub that operates 24 h a day, as use of telehealth for substance use disorder and specialist services in the ED is becoming more common [32, 33].

Rural ED providers were reluctant to refer eligible patients to RCPSI peers. While there was no discussion of the specific reasons, prior work has noted difficulties adapting interventions to rural areas when they are incompatible with provider experience [31]. Additionally, incompatibilities between peers’ workflow and established and longstanding processes within the ED without appropriate information and technology to support integration likely had some negative impact on implementation of the referral process [34]. Reluctance to initiate referrals could also be rooted in high levels of individual stigma toward substance users and services designed to assist them, which prior research has demonstrated to be common in rural communities [35–37]. For instance, Richard et al.’s [38] analysis of semi structured interviews conducted with rural Appalachian stakeholders, including healthcare professionals, demonstrated conservative abstinence-focused attitudes that stood in opposition to MOUD. It is possible stigmatizing attitudes influenced rural providers’ willingness to communicate with and make referrals to non-peer behavioral health staff, since peers were known by providers to be in recovery from a substance use disorder. More research should seek to understand peers as both recipients and potential mediators of stigmatizing attitudes among the providers they work alongside and how this might differ by the greater health services context. Regardless of the reason, the lack of patients resulted in the need to expand the role of some rural peers to ensure they were utilized. This meant assigning them to serve OUD patients in other hospital departments and through collaboration with community programs.

Despite low patient volume, rural vendors still expressed concern regarding inaccessibility of MOUD treatment for patients with whom peers worked. While not documented in the data, it is possible this concern reflected referrals for patients whom peers saw through their expanded job duties given ED referrals were low. Regardless, this resulted in a bottleneck for rural peers’ work that negatively impacted their ability to meet the third core function of ED-based peers [6]—connecting patients with MOUD or other treatment. Prior research has identified barriers to MOUD implementation and access as common in rural areas [39, 40]. Greater discomfort treating OUD patients is one possible reason for this shortage, and prior research comparing rural and urban physician attitudes toward treating OUD patients has demonstrated this likely has more to do with lower levels of training and experience among rural physicians [41]. Fortunately, there are interventions demonstrated to educate and support rural physicians, which hospitals could use to improve MOUD access in their areas. The Extensions for Community Healthcare Outcomes (ECHO) model offers education over a distance through video to facilitate case-based learning, and it has been shown to be a feasible model for expanding MOUD and other substance use treatments in rural communities [42, 43]. Another option, the hub-and-spoke approach, utilizes a network with a central anchor establishment (hub) with a full service array that supports secondary providers (spokes) with more limited services. The approach can improve provider confidence and has documented success improving rural system’s MOUD treatment capacity [44]; however, this does require the identification of a hub, which can be difficult in some rural communities [45]. If these two approaches are not feasible, rural hospitals can also consider developing internal MOUD capacity, as RV2 did.

The difficulty some rural peers experienced following-up with patients transported to urban hospitals was likely rooted in federal laws prohibiting the sharing of individual-level health information between providers without patient consent. The most well-known of these laws is the Health Insurance Portability and Accountability Act (HIPAA). However, Title 42 of the Code of Federal Regulations Part 2 (42 CFR Part 2) is a less known regulation among general healthcare providers that applies specifically to the release of information for behavioral health treatment. Prior work has demonstrated provider confusion related to 42 CRF Part 2 that is perceived to inhibit communication between providers, and this can in turn negatively impact care coordination [46]. While not documented in the data represented in this article, discussions from an informal learning collaborative in which RCPSI vendors participated [47] demonstrated 42 CFR Part 2 was a sticking point of confusion that limited communication between rural and urban hospitals. Fortunately, one of the main urban hospitals to which rural vendors transported patients participated in the collaborative, and they were able to work together to address some of the issues around 42 CFR Part 2.

At the RCPSI’s start, the state contractually required vendors to limit peer work to ED patients presenting with OUD. To ensure this, the state was initially only supporting peer work through cost reimbursement for services provided to eligible patients. This requirement limited rural hospitals because they did not not have the patient volume to support peer work under those terms. In recognition of this and other issues, the state changed the terms of the contracts to directly support peer salaries. Rural vendors were then able to adapt and allow peer work to expand beyond the confines of the ED. This highlights the need for state-wide initiatives to recognize contextual differences between rural and urban locations when initiating contracts, as well as understanding that rural programs should likely not be evaluated on the exact same terms as their urban counterparts [31]. Also connected to funding, rural vendors faced more unexpected costs over time related to the need to support MOUD providers and implement needed technology, and prior research has identified similar barriers to rural substance use disorder treatment [39].

Regarding limitations, interview data only represent three snapshots in time for each vendor. However, the data do provide a longitudinal picture of the first year of implementation and were conducted at key time points for the project. As such, the analysis demonstrates how implementation determinants evolved over time and how rural vendors were faced with greater challenges despite expectations similar to those of urban vendors at T1 interviews. The focus on 10 sites, only 4 of which were rural, has implications for applicability of the findings to other contexts; however, the sample is larger than the minimum recommended for making strong comparisons between cases [25, 26]. Additionally, the compatibility of findings with the broader rural-focused OUD literature strengthens their transferability [25, 26, 48] to other situations and thus their potential ability to inform implementation of similar programs. While the potential change in interview participants across time points for some sites might have impacted data reliability, the inclusion of primary designated implementation leaders in each interview provided some consistency, making it likely key issues were discussed. Many of the issues discussed in interviews cut across constructs. For instance, lack of appropriate technology to support the intervention could cut across intervention characteristics, inner setting climate, and unexpected costs related to the implementation process depending on how the issue is framed. While this could have led to difficulties tracing the exact nature of a particular implementation challenge, the longitudinal nature of the data helped us to better understand the nature of specific issues raised by tracing how they unfolded over time. Finally, it is important to note that the absence of the discussion of a particular determinant within a site or among a group of sites (e.g., urban, rural) does not mean it was necessarily absent; however, it does mean it was unlikely to have had as influential of an impact as those that were the focus of interview participants’ discussions.

## Conclusions

Secondary analysis of interviews from the RCPSI evaluation identified several issues related to program implementation that differed between rural and urban contexts. Such information is important given the majority of research on ED-based OUD interventions has been urban-focused to date. Most notably, findings demonstrate how rural hospitals were faced with greater challenges implementing ED-based peers that required flexible adaptations to originally intended plans. Funders should allow rural programs to have such flexibility when interventions they are implementing were developed in or for urban settings.

## Data Availability

Qualitative data are not available due to confidentiality concerns related to such a small sample.

## Abbreviations

CFIR: Consolidated Framework for Implementation Research
ED: Emergency department
MOUD: Medications for opioid use disorder
OUD: Opioid use disorder
RCPSI: Recovery Coach and Peer Support Initiative

## Footnotes

1 Instances where urban vendors discussed issues with rural sites were easy to identify and excluded from the analysis because the information provided was too few and far between to add substantially to the understanding of rural-urban differences.
